# Premenstrual Dysphoric Disorder Prevalence and Symptoms Across Age Groups: A Cross‐Sectional Study

**DOI:** 10.1111/1471-0528.18261

**Published:** 2025-07-07

**Authors:** Adriana Orcesi Pedro, Roberto Carmignani Verdade, Maura Gonzaga Lapa, Juliana Dinéia Perez Brandão, Vivienne Carduz Castilho

**Affiliations:** ^1^ Department of Gynecology and Obstetrics Universidade Estadual de Campinas Campinas Brazil; ^2^ Libbs Farmacêutica Ltda Scientific Medical Division São Paulo Brazil; ^3^ Medical Affairs Division PostdocLibbs Farmacêutica Ltda São Paulo Brazil

**Keywords:** Brazilian population, premenstrual dysphoric disorder, psychoemotional symptoms, somatic symptoms

## Abstract

**Objective:**

To estimate the prevalence and symptom severity of premenstrual dysphoric disorder (PMDD) in Brazilian women according to age groups, and to conduct an association analysis between psychoemotional and somatic symptoms.

**Design:**

Cross‐sectional study.

**Setting:**

303 private clinics across Brazil.

**Population:**

45 160 women aged 20–49 years.

**Methods:**

Self‐report questionnaire regarding the prevalence and intensity of premenstrual symptoms. Statistical analysis performed using Pearson's chi‐square test and Poisson regression and multiple logistic regression.

**Main Outcome Measures:**

Prevalence and intensity of somatic and psychoemotional premenstrual symptoms.

**Results:**

Prevalence of PMDD was 3.57% (95% CI: 3.40–3.75). Psychoemotional symptoms were more prevalent than somatic symptoms, with anxiety/tension (99.9%) and irritability/anger (99.8%) being the most frequently reported symptoms. Weight gain (92.5%) and edema (92.1%) were the most prevalent somatic symptoms. Anxiety/tension and headache occurred independently of other symptoms. Binge eating was associated with weight gain (OR = 2.77, 95% CI [2.11, 3.62]), acne (OR = 2.37, 95% CI [1.79, 3.10]), immunoallergic exacerbations (OR = 1.81, 95% CI [1.26, 2;60]) and edema (OR—0.74, 95% CI [0.55, 0.97]). Affective lability was associated with immunoallergic exacerbations (OR = 1.49, 95% CI [1.16, 1.91]) and mastalgia (OR = 1.29, 95% CI [1.02, 1.63]). Depression was associated with acne (OR = 0.72, 95% CI [0.57, 0.89]) and weight gain (OR = 0.77, 95% CI [0.61, 0.96]).

**Conclusions:**

The prevalence of PMDD was consistent with other population studies. Psychoemotional symptoms were more prevalent. Association analysis provided new insights into premenstrual symptomatology.

## 1 Introduction

Premenstrual symptoms can manifest at any point after menarche and considerably affect women's quality of life, mental health and personal and work relationships, especially in severe cases [1]. Premenstrual symptoms can be divided into somatic and psychological components. Somatic symptoms include abdominal pain, cramps, nausea, breast pain and swelling, whereas psychological symptoms are further divided into affective (e.g., irritability, anxiety, depressive mood), cognitive (e.g., difficulty in concentrating, memory loss), neurovegetative (e.g., low libido, insomnia, hypersomnia, fatigue, lack of energy, increased appetite) and behavioural (e.g., withdrawal from daily activities) symptoms. These symptoms have a clear temporal relationship with the premenstrual period and cause significant distress that interferes with functionality [2].

The fifth edition of the Diagnostic and Statistical Manual of Mental Disorders classifies premenstrual symptoms as depressive disorders under the name “premenstrual dysphoric disorder” (PMDD). There must be a minimum of five symptoms, one of which is a core symptom: affective lability, irritability, depressed mood and anxiety. The term “premenstrual syndrome” (PMS) is applicable to cases in which there are insufficient symptoms of PMDD, which usually occur with less severity.

Up to 91% of women experience at least one premenstrual symptom [3]. A meta‐analysis with a sample of 18 803 individuals from different countries found a pooled prevalence of 47.8% (95% CI = 32.6–62.9) for PMS. Prevalence varied greatly depending on the population studied, with relatively low rates found in countries such as Switzerland (10%, 95% CI = 9.1–10.9) and France (12%, 95% CI = 10.8–13.2) and high rates in countries such as Iran (98.2%, 95% CI = 96.7–99.7) and Nigeria (85%, 95% CI = 80.1–89.9) [4]. A recent meta‐analysis on the prevalence of PMDD, with 50 659 participants, reported a pooled prevalence of 3.2% (95% CI = 1.7–5.9) for confirmed diagnosis (defined as prospective monitoring of symptoms for at least two menstrual cycles) and 7.7% (95% CI = 5.3–8.4) for provisional diagnosis (defined as any other diagnostic method) [5]. In Brazil, a multicenter study conducted telephone interviews with a sample of 1053 women and reported a prevalence of 60.3% for premenstrual symptoms [6]. A cross‐sectional population‐based study on self‐reported symptoms in 1395 women indicated a similar percentage; however, the prevalence decreased to 25.2% when stricter diagnostic criteria were applied [7]. Silva et al. analysed the Pelotas cohort and found that 13.4% and 5.8% of 2082 eligible women experienced moderate and severe PMS symptoms, respectively [8]. The most recent prevalence study in Brazil analysed data from 1115 university students, aged between 20.1 and 22 years, and found that 11.1% (95% CI = 9.3–13.0) had criteria for PMDD [9]. There are no recent studies assessing the prevalence of PMDD in Brazilian women. Most were published more than 10 years ago or analysed a specific population.

The present study primarily aimed to estimate the prevalence of PMDD based on self‐reported somatic and psychoemotional symptoms. Its secondary objectives were to estimate the prevalence and severity of each premenstrual symptom by age and to conduct an association analysis between psychoemotional and somatic symptoms. This is a secondary analysis from a previous study that evaluated the prevalence, intensity and regional distribution of PMS in the same population.

This study will improve knowledge and awareness of a disorder that significantly impacts the quality of life of women of reproductive age. The data from this study may guide what should be valued in the clinical evaluation of premenstrual symptoms.

## 2 Methods

A self‐report questionnaire was offered from February 2019 to March 2020 to 56 948 women, of which 49 029 aged 20–49 years met the inclusion criteria and were selected.

The prevalence of PMDD in the selected sample was calculated using the definition derived from the American College of Obstetricians and Gynaecologists and the Royal College of Obstetricians and Gynaecologists [10]. This study included women who answered “Bothers a lot” to the question “How much do the mentioned symptoms interfere with your daily life?” and who had at least five severe symptoms:

- At least one of the four core symptoms: affective lability, irritability and anger, depression and sadness or anxiety and tension.
- At least one of other psychoemotional symptoms: decreased interest in routine activities, difficulty in concentrating or binge eating.
- Any somatic symptoms counted as one symptom (might not have any).

Women were selected from 303 private clinics located in large‐ and medium‐sized cities across 22 Brazilian states in all five regions of Brazil, using a convenience sample approach. Most clinics offered multiple specialties, not only gynaecological care, and the participants were also selected among women who were accompanying patients. They were invited to participate in the study while waiting in a clinic waiting room. The invitation came through an electronic device, such as a cell phone or tablet, as soon as they requested access to the clinic's wireless network. Information about the content and purpose of the study was provided to the participants, who were subsequently invited after signing a digital consent form and directed to complete a questionnaire adapted from the Brazilian‐validated version of the Premenstrual Symptoms Screening Tool [11, 12]. The average time to complete the questionnaire was 5–10 min. Data were collected from a database containing information stored by market research programmes and anonymised to ensure data security and confidentiality. All collected data were stored on the servers of the outsourcing company Wispot. The study protocol was approved by the INVITARE Research Ethics Committee—no. 8098 under registration number 33794520.1.0000.8098. Informed consent was obtained from all participants. Date of approval: January 19th, 2021.

### 2.1 Statistical Analysis

The prevalence of PMDD and its corresponding 95% confidence interval (CI) were calculated. Frequency tables were created for categorical variables using absolute frequency (*n*) and percentage (%) to describe the profile of participants with PMDD based on the study variables. Answers to each question were compared among the age groups using Pearson's chi‐square test. The level of significance was set at 5% (or 95% CI). The number of symptoms was compared among the age groups using Poisson regression, with the number of symptoms being the dependent variable and the age groups being the independent variable. There was no adjustment for covariates and there was no missing data, since only fully completed questionnaires were used. The association between psychoemotional and somatic symptoms was assessed using multiple logistic regression models. All statistical analyses were performed using SAS software version 9.4 (SAS Institute Inc., Cary, NC, USA).

## 3 Results

**FIGURE 1:**
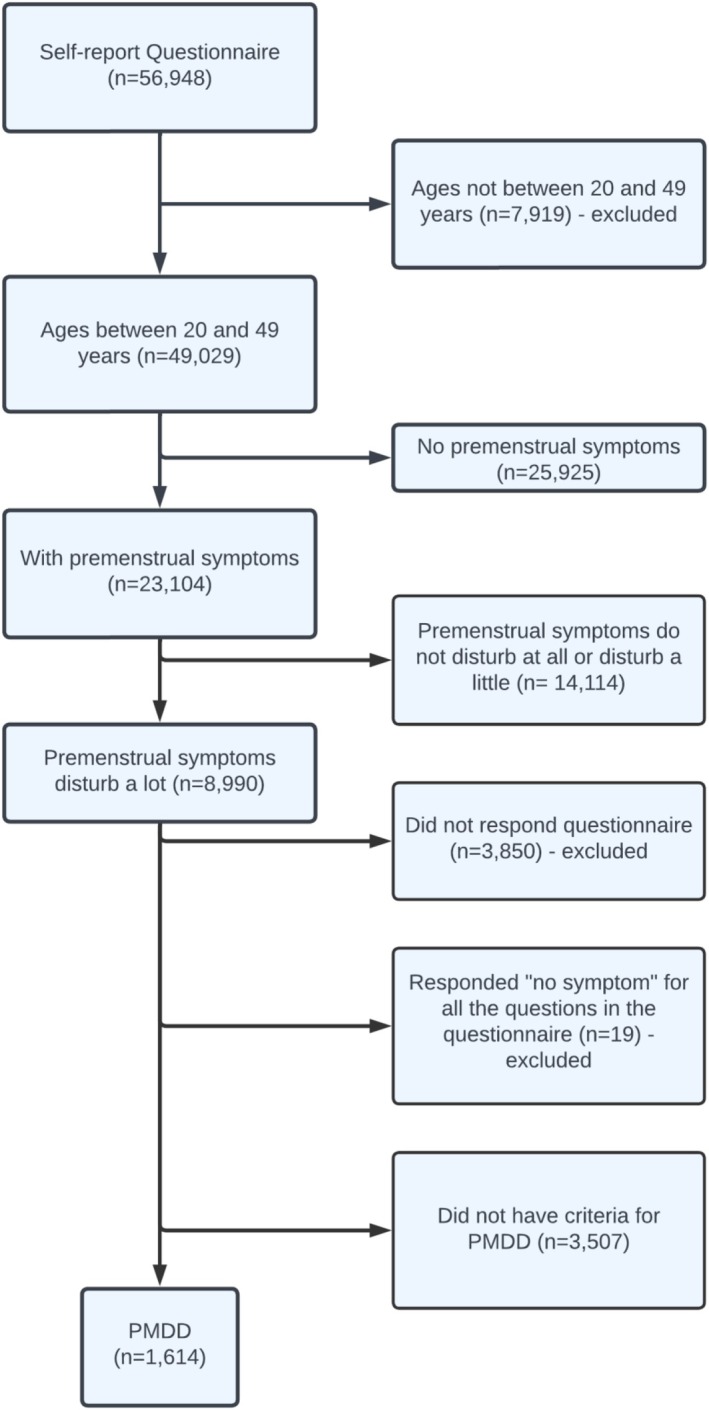
Flowchart of participant selection.

Out of 56 948 women, 49 029 aged 20–49 years were selected, and 3850 did not adequately complete the questionnaire were excluded. Additionally, 19 women who answered “no symptom” to all questions after reporting that they had premenstrual symptoms were excluded. A total of 1614 women experienced PMDD symptoms, resulting in a prevalence of 3.57% (95% CI: 3.40–3.75). Symptom analysis was conducted only on women who met criteria for PMDD (Figure 1).

According to age group, 694 (43.0%) were 20–29 years, 666 (41.3%) were 30–39 years and 254 (15.7%) were 40–49 years old. With respect to regional distribution, 1106 (68.5%), 203 (12.6%), 160 (9.9%), 85 (5.3%) and 60 (3.7%) women were from the Southeast, Northeast, South, Midwest and North Regions, respectively (Table S1).

Psychoemotional symptomatology was the most prevalent, with all psychoemotional symptoms being more prevalent than somatic symptoms even when stratified by age. Anxiety and tension and irritability and anger were the most prevalent symptoms at any intensity and age, with 99.9% (95% CI [99.9%–99.9%]) and 99.8% (95% CI [99.8%–99.8%]), respectively, and at severe intensity with 86.7% (95% CI [86.1%–87.2%]) and 84.5% (95% CI [83.9%–85.1%]). Weight gain and edema were the most prevalent somatic symptoms at any intensity, with 92.5% (95% CI [92.2%–92.8%]) and 92.1% (95% CI [91.8%–92.5%]), respectively (Table 1).

**TABLE 1:** Prevalence of total and severe PMDD symptoms by age group (*n* = 1614).

Prevalence of total and severe PMDD symptoms by age group—*n* (%) (95% CI)		Age group (years)			Total	*p*
		20–29	30–39	40–49	(*n* = 1614)	
		(*n* = 694)	(*n* = 666)	(*n* = 254)		
Anxiety and tension	Total	693 (99.9%) (99.8%, 99.9%)	666 (100.0%) (100%, 100%)	254 (100.0%) (100%, 100%)	1613 (99.9%) (99.9%, 99.9%)	0.515
	Severe	582 (84.0%) (83.1%, 84.9%)	581 (87.2%) (86.4%, 88%)	235 (92.5%) (91.7%, 93.3%)	1398 (86.7%) (86.1%, 87.2%)	0.002
Irritability and anger	Total	693 (99.9%) (99.8%, 99.9%)	664 (99.7%) (99.7%, 99.7%)	254 (100.0%) (100%, 100%)	1611 (99.8%) (99.8%, 99.8%)	0.604
	Severe	545 (78.6%) (77.5%, 79.8%)	582 (87.7%) (86.9%, 88.4%)	234 (92.1%) (91.3%, 93%)	1361 (84.5%) (83.9%, 85.1%)	< 0.001
Depression and sadness	Total	684 (98.6%) (98.5%, 98.7%)	662 (99.4%) (99.4%, 99.4%)	254 (100.0%) (100%, 100%)	1600 (99.1%) (99.1%, 99.2%)	0.066
	Severe	470 (68.7%) (67.4%, 70%)	478 (72.2%) (70.9%, 73.5%)	174 (68.5%) (66.3%, 70.7%)	1112 (70.1%) (69.3%, 71%)	0.311
Decreased interest in routine activities	Total	684 (98.6%) (98.5%, 98.7%)	660 (99.1%) (99%, 99.2%)	250 (98.4%) (98.2%, 98.6%)	1594 (98.8%) (98.7%, 98.8%)	0.581
	Severe	532 (77.8%) (76.6%, 78.9%)	469 (71.1%) (69.7%, 72.4%)	180 (72.0%) (69.9%, 74.1%)	1181 (74.1%) (73.3%, 74.9%)	0.014
Difficulty in concentrating	Total	676 (97.4%) (97.2%, 97.6%)	649 (97.4%) (97.3%, 97.6%)	247 (97.2%) (96.9%, 97.6%)	1572 (97.4%) (97.3%, 97.5%)	0.985
	Severe	304 (45.0%) (43.7%, 46.2%)	307 (47.3%) (46%, 48.6%)	115 (46.6%) (44.4%, 48.7%)	726 (46.2%) (45.3%, 47%)	0.69
Binge eating	Total	677 (97.6%) (97.4%, 97.7%)	649 (97.4%) (97.3%, 97.6%)	244 (96.1%) (95.6%, 96.5%)	1570 (97.3%) (97.1%, 97.4%)	0.432
	Severe	570 (84.2%) (83.3%, 85.1%)	532 (82.0%) (80.9%, 83%)	174 (71.3%) (69.1%, 73.5%)	1276 (81.3%) (80.6%, 82%)	< 0.001
Affective lability	Total	665 (95.8%) (95.5%, 96.1%)	642 (96.4%) (96.1%, 96.7%)	248 (97.6%) (97.4%, 97.9%)	1555 (96.3%) (96.2%, 96.5%)	0.417
	Severe	320 (48.1%) (46.8%, 49.4%)	345 (53.7%) (52.3%, 55.1%)	162 (65.3%) (63%, 67.6%)	827 (53.2%) (52.3%, 54.1%)	< 0.001
Total psychoemotional symptoms	Total	100%	100%	100%	100%	—
	Severe	100%	100%	100%	100%	—
Weight gain	Total	610 (87.9%) (87.2%, 88.6%)	640 (96.1%) (95.8%, 96.4%)	243 (95.7%) (95.2%, 96.2%)	1493 (92.5%) (92.2%, 92.8%)	< 0.001
	Severe	314 (51.5%) (50.1%, 52.9%)	369 (57.7%) (56.2%, 59.1%)	141 (58.0%) (55.7%, 60.4%)	824 (55.2%) (54.3%, 56.1%)	0.056
Edema	Total	638 (91.9%) (91.4%, 92.5%)	610 (91.6%) (91%, 92.2%)	239 (94.1%) (93.4%, 94.8%)	1487 (92.1%) (91.8%, 92.5%)	0.407
	Severe	246 (38.6%) (37.4%, 39.7%)	257 (42.1%) (40.9%, 43.4%)	90 (37.7%) (35.8%, 39.5%)	593 (39.9%) (39.1%, 40.6%)	0.325
Headache	Total	617 (88.9%) (88.2%, 89.6%)	627 (94.1%) (93.7%, 94.6%)	241 (94.9%) (94.3%, 95.5%)	1485 (92.0%) (91.7%, 92.4%)	< 0.001
	Severe	301 (48.8%) (47.4%, 50.2%)	380 (60.6%) (59.2%, 62.1%)	135 (56.0%) (53.7%, 58.3%)	816 (54.9%) (54%, 55.9%)	< 0.001
Acne/skin oiliness	Total	663 (95.5%) (95.2%, 95.8%)	601 (90.2%) (89.6%, 90.9%)	218 (85.8%) (84.4%, 87.2%)	1482 (91.8%) (91.5%, 92.2%)	< 0.001
	Severe	380 (57.3%) (55.9%, 58.7%)	250 (41.6%) (40.3%, 42.9%)	71 (32.6%) (30.9%, 34.2%)	701 (47.3%) (46.4%, 48.2%)	< 0.001
Mastalgia	Total	588 (84.7%) (83.8%, 85.6%)	628 (94.3%) (93.9%, 94.7%)	235 (92.5%) (91.7%, 93.3%)	1451 (89.9%) (89.5%, 90.3%)	< 0.001
	Severe	183 (31.1%) (30.2%, 32.1%)	246 (39.2%) (38%, 40.3%)	86 (36.6%) (34.8%, 38.4%)	515 (35.5%) (34.8%, 36.2%)	0.013
Immunoallergic exacerbations	Total	605 (87.2%) (86.4%, 88%)	592 (88.9%) (88.2%, 89.6%)	230 (90.6%) (89.5%, 91.6%)	1427 (88.4%) (87.9%, 88.9%)	0.314
	Severe	181 (29.9%) (29%, 30.8%)	152 (25.7%) (24.9%, 26.5%)	54 (23.5%) (22.4%, 24.6%)	387 (27.1%) (26.6%, 27.7%)	0.102
Total somatic symptoms	Total	694 (100%) (100%, 100%)	666 (100%) (100%, 100%)	253 (99.9%) (99.6%, 99.6%)	1613 (99.9%) (99.9%, 99.9%)	0.067
	Severe	655 (94.4%) (94%, 94.8%)	619 (92.9%) (92.4%, 93.4%)	231 (90.9%) (89.9%, 91.9%)	1505 (93.2%) (92.9%, 93.5%)	0.161

*Note:* Total = prevalence of any intensity (mild, moderate and severe)/Severe = prevalence of severe intensity.

When only severe symptoms were selected, the 20–29‐year age group exhibited a significantly lower prevalence of irritability and anger (78.6%, 95% CI [77.5%–79.8%]), headache (48.8%, 95% CI [47.4%–50.2%]), affective lability (48.1%, 95% CI [46.8%–49.4%]) and mastalgia (31.1%, 95% CI [30.2%–32.1%]) than the older age groups. The prevalence of loss of interest in daily activities (77.8%, 95% CI [76.6%–78.9%]) and acne (57.3%, 95% CI [55.9%–58.7%]) was higher in the 20–29‐year age group than in the older age groups (Table 1).

Analysis of the number of symptoms in different age groups revealed that the total number of moderate and severe psychoemotional symptoms did not differ among the age groups. The average number of severe symptoms, including both psychoemotional and somatic symptoms, also did not differ among the groups. The younger group had an average of 7.1 severe symptoms, whereas the 30–39‐year and 40–49‐year age groups had 7.4 and 7.3 severe symptoms, respectively (*p* = 0.079; Table S2). However, the number of moderate and severe somatic symptoms was significantly lower in the 20–29‐year group. Overall, 28.5% of those women had six symptoms, while 37.5% and 33.9% of women in the 30–39‐year and 40–49‐year age groups, respectively, had the same number of moderate and severe somatic symptoms (*p* < 0.001) (Table 2).

**TABLE 2:** Number of moderate and severe psychoemotional and somatic PMDD symptoms by age group (*n* = 1614).

	20–29 years (*n* = 694)	30–39 years (*n* = 666)	40–49 years (*n* = 254)	Total (*n* = 1614)
Number of moderate and severe psychoemotional PMDD symptoms, *n* (%)				
0–3	0 (0.0%)	0 (0.0%)	0 (0.0%)	0 (0.0%)
4	20 (2.9%)	17 (2.6%)	4 (1.6%)	41 (2.5%)
5	108 (15.6%)	74 (11.1%)	21 (8.3%)	203 (12.6%)
6	231 (33.3%)	157 (23.6%)	64 (25.2%)	452 (28.0%)
7	335 (48.3%)	418 (62.8%)	165 (65.0%)	918 (56.9%)
Median (Q1–Q3)	6 (6–7)	7 (6–7)	7 (6–7)	7 (6–7)
*p* (Poisson regression for psychoemotional symptoms and age group) = 0.222				
Number of moderate and severe somatic PMDD symptoms, *n* (%)				
0	5 (0.7%)	1 (0.2%)	3 (1.2%)	9 (0.6%)
1	29 (4.2%)	15 (2.3%)	5 (2.0%)	49 (3.0%)
2	72 (10.4%)	57 (8.6%)	19 (7.5%)	148 (9.2%)
3	114 (16.4%)	85 (12.8%)	35 (13.8%)	234 (14.5%)
4	125 (18.0%)	115 (17.3%)	34 (13.4%)	274 (17.0%)
5	151 (21.8%)	143 (21.5%)	72 (28.3%)	366 (22.7%)
6	198 (28.5%)	250 (37.5%)	86 (33.9%)	534 (33.1%)
Total	694 (100.0%)	666 (100.0%)	254 (100.0%)	1614 (100.0%)
Median (Q1–Q3)	5 (3–6)	5 (4–6)	5 (4–6)	5 (3–6)
**p (Poisson regression for somatic symptoms and age group) < 0.001**. ***p* (20–29 vs. 30–39) = 0.004/*p* (20–29 vs. 40–49) = 0.046/** *p* (30–39 vs. 40–49) = 0.868.				

Bold values are the statistically significant *p* values (*p* < 0.05).

The association analysis between psychoemotional and somatic symptoms (Table 3) showed that irritability and anger were associated with edema (OR = 0.64, 95% CI [0.48, 0.87]) and acne (OR = 0.56, 95% CI [0.42, 0.74]). Depression was associated with acne (OR = 0.72, 95% CI [0.57, 0.89]) and weight gain (OR = 0.77, 95% CI [0.61, 0.96]). Decreased interest in routine activities was associated with edema (OR = 1.79, 95% CI [1.38, 2,31]). Binge eating was associated with weight gain (OR = 2.77, 95% CI [2.11, 3.62]), edema (OR = 0.74, 95% CI [0.55, 0.97]), acne (OR = 2.36, 95% CI [1.79, 3.10]) and immunoallergic exacerbations (OR = 1.81, 95% CI [1.26, 2.60]). Affective lability was associated with immunoallergic exacerbations (OR = 1.49, 95% CI [1.16, 1.91]) and mastalgia (OR = 1.29, 95% CI [1.02, 1.63]). Anxiety and headache occurred independently of other symptoms.

**TABLE 3:** Association between psychoemotional and somatic PMDD symptoms.

Psychoemotional symptoms		Overall average	Somatic symptoms					
			Mastalgia	Headache	Weight gain	Edema	Acne/skin oiliness	Immunoallergic exacerbations
Irritability and anger	OR (95% CI)	7.98 (6.04, 10.54)	0.92 (0.67, 1.27)	1.19 (0.88, 1.58)	0.95 (0.71, 1.26)	**0.65 (0.48, 0.87)**	**0.56 (0.42, 0.74)**	1.1 (0.78 1.53)
	Probability	88.90%	88.00%	90.40%	88.30%	**83.90%**	**81.80%**	89.80%
	*p*		0.620	0.250	0.721	**0.004**	**0.000**	0.575
Anxiety and tension	OR (95% CI)	7.19 (5.4, 9.58)	1.26 (0.89, 1.79)	0.82 (0.60, 1.10)	1.14 (0.84, 1.54)	0.79 (0.57, 1.08)	0.75 (0.56, 1.01)	1.39 (0.94, 2.02)
	Probability	87.80%	90.10%	85.50%	89.20%	85.10%	84.40%	90.90%
	*p*		0.140	0.185	0.197	0.380	0.153	0.063
Depression and sadness	OR (95% CI)	3.04 (2.46, 3.77)	0.98 (0.75, 1.26)	0.93 (0.73, 1.15)	**0.77 (0.61, 0.96)**	0.98 (0.77, 1.24)	**0.72 (0.57, 0.89)**	1.23 (0.93, 1.60)
	Probability	75.30%	74.90%	73.80%	**70.20%**	74.90%	**68.50%**	78.90%
	*p*		0.869	0.500	**0.024**	0.887	**0.003**	0.131
Decreased interest in routine activities	OR (95% CI)	2.01 (1.62, 2.49)	1.26 (0.96, 1.66)	1.17 (0.92, 1.47)	0.89 (0.70, 1.12)	**1.79 (1.38, 2.31)**	1.16 (0.91, 1.45)	0.85 (0.63, 1.12)
	Probability	66.80%	71.80%	70.20%	64.10%	**78.30%**	69.90%	63.00%
	*p*		0.090	0.186	0.319	**0.000**	0.214	0.246
Binge eating	OR (95% CI)	1.7 (1.35, 2.13)	0.93 (0.68, 1.24)	1.13 (0.87, 1.46)	**2.77 (2.11, 3.62)**	**0.74 (0.55, 0.97)**	**2.36 (1.79, 3.10)**	**1.81 (1.26, 2.60)**
	Probability	62.90%	61.10%	65.70%	**82.40%**	**55.50%**	**80.00%**	**75.40%**
	*p*		0.616	0.354	**0.000**	**0.031**	**0.000**	**0.001**
Affective lability	OR (95% CI)	0.86 (0.71, 1.04)	**1.29 (1.02, 1.63)**	1.09 (0.88, 1.33)	0.97 (0.78, 1.18)	1.08 (0.87, 1.34)	0.94 (0.76, 1.15)	**1.49 (1.16, 1.91)**
	Probability	46.20%	**52.60%**	48.20%	45.30%	48.20%	44.60%	**56.10%**
	*p*		**0.032**	0.440	0.739	0.465	0.551	**0.002**
Difficulty in concentrating	OR (95% CI)	0.61 (0.5, 0.74)	**1.44 (1.14, 1.82)**	1 (0.81, 1.23)	0.87 (0.70, 1.07)	**0.22** **1.25 (1.01, 1.56)**	0.14 1.15 (0.93, 1.41)	**0.4** **1.5 (1.17, 1.91)**
	Probability	37.90%	**46.90%**	38.00%	34.70%	**43.30%**	41.20%	**47.80%**
	*p*		**0.002**	0.965	0.192	**0.045**	0.186	**0.001**

Statistically significant *p* values are **bold/**associated symptoms are highlighted in grey/probability = Exp(Y′)/(1 + Exp(Y′)).

Most women (77.2%) were willing to use oral contraception as a therapeutic alternative for PMDD. The younger group showed even greater interest (83.9%), which was statistically significant when compared to the 30–39‐year group (72.7%) and 40–49‐year group (70.9%) (*p* < 0.001; Table 4).

**TABLE 4:** Distribution of women according to age group regarding their willingness to take contraceptives as an option to treat PMDD.

Willingness to take contraceptives, *n* (%)	Age groups (years)			
	20–29 (*n* = 694)	30–29 (*n* = 666)	40–49 (*n* = 254)	Total (*n* = 1614)
No	112 (16.1%)	182 (27.3%)	74 (29.1%)	368 (22.8%)
Yes	582 (83.9%)	484 (72.7%)	180 (70.9%)	1246 (77.2%)
Total	694 (100.0%)	666 (100.0%)	254 (110.0%)	1614 (100.0%)
*p* (chi‐squared for willingness to take contraceptives * profile) < 0.001				
		Difference		
***p* (20–29 vs. 30–39) < 0.001**		**−11.02%**		
***p* (20–29 vs. 40–49) < 0.001**		**−13.0%**		
*p* (30–39 vs. 40–49) = 0.585		−1.8%		

Bold values are the statistically significant *p* values (*p* < 0.05).

## 4 Discussion

### 4.1 Main Findings

The prevalence of PMDD found in this study is consistent with that reported by other investigations, in which the percentages varied from 1.8% to 5.8% [2, 3, 5], including Brazilian studies [8]. This result is similar to the pooled prevalence of confirmed diagnosis found in the largest and most recent systematic review on the prevalence of PMDD [5]. The high prevalence of psychoemotional symptoms is also consistent with the findings of other studies [6, 7]. A previous study based on the same population found that 38.91% met criteria for PMS [13].

Although affective lability and depression/sadness are core symptoms [2], they were not among the four most common severe symptoms. Depression/sadness ranked as the fifth most prevalent symptom, behind decreased interest in routine activities. Affective lability was the eighth most prevalent severe symptom, behind weight gain and headache. Further studies should focus on reassessing the prevalence of these core symptoms and confirming or ruling out their importance in diagnosis. The use of mathematical models to analyse clinical data could prove useful for this task. A 2012 study used mathematical models to propose a method of diagnosing PMS (not PMDD) using fewer number of symptoms and showed that depression and social withdrawal were the most important predictors of PMS [14]. The use of similar methods to analyse PMDD symptoms in a larger population could aid in establishing new and more accurate diagnostic criteria. It would also be interesting to evaluate the specificity of each symptom for diagnosis, as most of these symptoms could also be present in different disorders, such as borderline personality disorder, bipolar disorder and depression, or be premenstrual exacerbations of these disorders, increasing the difficulty in making an accurate diagnosis [15, 16, 17, 18].

We expected to observe fewer symptoms in the 40–49‐year‐old group because severe symptoms have been reported by epidemiological studies to be more prevalent in younger women [9, 19]. No statistically significant difference was found among the age groups with respect to the total number of severe symptoms. Additionally, the number of moderate and severe psychoemotional symptoms did not significantly differ, however our analysis showed that somatic symptoms were less prevalent in the younger age group. It is worth discussing that although all participants reported menstruation, the frequency of cycles was not investigated. It is possible that some participants in the 40–49‐year‐old group were in the menopausal transition, a period in which the menstrual cycle starts to lengthen and perimenopausal symptoms may arise, a few of which may overlap with premenstrual symptoms [20]. Symptoms such as depression and anxiety may worsen during the menopausal transition in women with PMS or with more symptomatic menstruation during early life [21, 22, 23]. In this study, the only symptoms that were more prevalent in the 40–49‐year‐old group were anxiety/tension and affective lability.

Regarding treatment, more women in the 20–29‐year age group were willing to use contraceptives as treatment for premenstrual symptoms. This age group could benefit from contraception when necessary; if this is the chosen method, these medications would be worth considering. The slightly higher resistance to contraceptive use as treatment for PMDD may have arisen from the misconception that these medications can only be used for birth control or that hormonal treatments may pose a greater risk [24]. Older patients may benefit from educational programs on contraceptive knowledge.

### 4.2 Strengths and Limitations

A limitation of this study is that the symptoms were self‐reported using a screening method with high sensitivity but low specificity. This method was selected because of the large number of participants in this cross‐sectional study. Despite being more accurate, longitudinal interviews are likely to result in loss of participants. Also, the lack of a clinical diagnosis may have permitted the inclusion of women with medical conditions that might contribute to the symptoms. Additionally, participants were selected in a private clinic setting. Although most clinics had multiple specialties, not just gynaecological care, and participants were selected among companions, this may have resulted in a bias toward women with more severe symptoms and with a higher socio‐economical level, while excluding those who relied on public health services. Also, the digital questionnaire may have favoured women who were more familiar with technology, indirectly also favouring women with a higher socio‐economical level. As this is a cross‐sectional study, the association analysis does not establish a causal relationship between symptoms. There was no other data collected on sociodemographic or socio‐economical factors.

### 4.3 Interpretation

The prevalence of psychoemotional symptoms in women reflects the impact of PMDD on mental health. Although our analysis did not aim to establish causality, some proportional relationships, such as between binge eating and weight gain, are easier to understand [25], whereas others, such as difficulty in concentrating and immunoallergic exacerbations, are not easily explained. The lack of association was also notable. There was no significant association between anxiety/tension or headache and psychoemotional symptoms. Although this study was not designed to establish causal relationships between these symptoms or to gain a better understanding of their relationships, these findings may be useful for future research.

## 5 Conclusion

An understanding of the epidemiology of PMDD in this sample of Brazilian women and the symptomatic profiles in different age groups can guide the development of more effective and specific diagnostic and treatment protocols for each population. Because menstrual disorders have characteristics that fit into two major specialties (namely, psychiatry and gynaecology), they do not often receive due attention from health professionals, resulting in incomplete investigations or ineffective treatment. For a psychiatrist's clinical practice, knowing and investigating the patterns of symptom changes during the menstrual period, which are routinely performed by a gynaecologist, can help differentiate the aetiology of these symptoms in patients, possibly resulting in different therapeutic proposals. Gynaecologists may benefit from a better knowledge of therapeutic measures for these disorders, such as the use of antidepressants and non‐pharmacological therapeutic techniques. Although no evidence supports the superiority of a specific drug type, there are differences in side‐effect profiles between selective serotonin reuptake inhibitor antidepressants and oral contraceptives, which are both considered the first‐line treatments [10, 26]. By understanding the most prevalent symptoms and treatment expectations of women, the most appropriate treatment can be selected. This reduces the time required to achieve the desired response and minimises the impact of illness on their lives.

## Author Contributions

Adriana O. Pedro contributed to design, data analysis, wrote and reviewed the manuscript. Roberto Carmignani Verdade contributed to data analysis and wrote the manuscript. Juliana D. P. Brandão contributed to data analysis and revision of the manuscript. Maura G. Lapa contributed to the statistical and data analysis of the manuscript. Vivienne C. Castilho contributed to the design and revision of the manuscript. All authors discussed the results and contributed to the final manuscript.

## Ethics Statement

The study protocol was approved by the INVITARE Research Ethics Committee—n^o^ 8098 under registration number 33794520.1.0000.8098.

## Conflicts of Interest

Each author has confirmed compliance with the journal's requirements for authorship.

## Supporting information


**Table S1.** Regional distribution according to age group.

**Table S2.** Number of severe intensity symptoms by age group.

## Data Availability

The data can be accessed and shared when formally requested. The authors are committed to ethical standards and legal requirements.
