# Supplementary material for: Premenstrual Dysphoric Disorder Prevalence and Symptoms Across Age Groups: A Cross‐Sectional Study
Source: BJOG. 2025 Jul 7;132(11):1596–605. doi: 10.1111/1471-0528.18261 (PMC12411650; doi:10.1111/1471-0528.18261)
Supplement: Supplementary file 1 — Table S1. Regional distribution according to age group. [file BJO-132-1596-s002.docx]

**SUPPORTING INFORMATION**

**TABLE S1** Regional distribution according to age group

| **Region - n (%)** | **20 – 29**  **(n=694)** | **30 – 39**  **(n = 666)** | **40 - 49**  **(n = 254)** | **TOTAL**  **(n=1,614)** |
| --- | --- | --- | --- | --- |
| Midwest | 41 (5.9%) | 30 (4.5%) | 14 (5.5%) | 85 (5.3%) |
| North | 30 (4.3%) | 20 (3.0%) | 10 (3.9%) | 60 (3.7%) |
| Northeast | 95 (13.7%) | 80 (12.0%) | 28 (11.0%) | 203 (12.6%) |
| South | 79 (11.4%) | 63 (9.5%) | 18 (7.1%) | 160 (9.9%) |
| Southeast | 449 (64.7%) | 473 (71.0%) | 184 (72.4%) | 1,106 (68.5%) |
