# Supplementary material for: Premenstrual Dysphoric Disorder Prevalence and Symptoms Across Age Groups: A Cross‐Sectional Study
Source: BJOG. 2025 Jul 7;132(11):1596–605. doi: 10.1111/1471-0528.18261 (PMC12411650; doi:10.1111/1471-0528.18261)
Supplement: Supplementary file 2 — Table S2. Number of severe intensity symptoms by age group. [file BJO-132-1596-s001.docx]

**SUPPORTING INFORMATION**

**TABLE S2** Number of severe intensity symptoms by age group

| **Number severe symptoms – n (%)** | **20 - 29**  **(n=694)** | **30 - 39**  **(n=666)** | **40 - 49**  **(n = 254)** | **TOTAL**  **(n=1,614)** |
| --- | --- | --- | --- | --- |
| 5 | 153 (22.0%) | 124 (18.6%) | 44 (17.3%) | 321 (19.9%) |
| 6 | 160 (23.1%) | 129 (19.4%) | 66 (26.0%) | 355 (22.0%) |
| 7 | 145 (20.9%) | 141 (21.2%) | 46 (18.1%) | 332 (20.6%) |
| 8 | 92 (13.3%) | 88 (13.2%) | 38 (15.0%) | 218 (13.5%) |
| 9 | 61 (8.8%) | 80 (12.0%) | 24 (9.4%) | 165 (10.2%) |
| 10 | 38 (5.5%) | 41 (6.2%) | 16 (6.3%) | 95 (5.9%) |
| 11 | 26 (3.7%) | 34 (5.1%) | 10 (3.9%) | 70 (4.3%) |
| 12 | 10 (1.4%) | 18 (2.7%) | 7 (2.8%) | 35 (2.2%) |
| 13 | 9 (1.3%) | 11 (1.7%) | 3 (1.2%) | 23 (1.4%) |
| Total | 694 (100.0%) | 666 (100.0%) | 254 (100.0%) | 1614 (100.0%) |
| Average  % above average | 7.1 | 7.4 | 7.3 | 7.3 |
|  | 34.0% | 40.8% | 38.6% | 37.5% |
| Median – Q1 – Q3 | 7 (6-8) | 7 (6-9) | 7 (6-8) | 7 (6-8) |
| p (Poisson regression for severe symptoms*age group) = 0.079 | | | | |
